# MPS VII – Extending the classical phenotype

**DOI:** 10.1016/j.ymgmr.2022.100922

**Published:** 2022-10-20

**Authors:** A. Oldham, N.J. Oxborrow, P. Woolfson, P. Jenkins, C. Gadepalli, J. Ashworth, A. Saxena, M. Rothera, C.J. Hendriksz, G. Tol, A. Jovanovic

**Affiliations:** a Mark Holland Metabolic Unit, Salford Royal NHS Foundation Trust, United Kingdom; b Salford Royal NHS Foundation Trust, United Kingdom; c Cardiology Department, Salford Royal NHS Foundation Trust, United Kingdom; d North West Congenital Heart Disease Partnership, Mark Holland Metabolic Unit, Salford Royal NHS Foundation Trust, Stott Lane, Salford, M6 8HD, United Kingdom; e Department of Ear, Nose and Throat, Salford Royal NHS Foundation Trust, United Kingdom; f Manchester Royal Eye Hospital, Manchester Foundation NHS Trust, United Kingdom; g Neurosurgery, Salford Royal NHS Foundation Trust, United Kingdom; h Royal Manchester Children's Hospital, United Kingdom; i University of Pretoria, Mark Holland Metabolic Unit, Salford Royal NHS Foundation Truist, Stott Lane, Salford, M6 8HD, United Kingdom

**Keywords:** Mucopolysaccharidosis VII, Sly syndrome, Case report, GUSB

## Abstract

Mucopolysaccharidosis VII (or Sly syndrome) is an autosomal recessive disorder characterised by a deficiency in the enzyme Beta-glucuronidase (*GUSB*). Partial degradation of glycosaminoglycans (GAGs); chondroitin sulfate (CS), dermatan sulfate (DS) and heparan sulfate (HS) results in the accumulation of these fragments in the lysosomes of many tissues, eventually leading to multisystem damage. In some cases, early diagnosis on clinical grounds alone can be difficult due to the extreme variability of the clinical presentation and disease progression. We present a case report of a 31-year-old male patient diagnosed with MPS VII at the age of 28, who multiple specialists saw without suspecting the diagnosis due to the unusual presentation. The patient presented with a history of developmental delay, scoliosis, kyphosis, corneal clouding, abnormal gait, short stature, hearing impairment, slightly coarse facial features and progressive deterioration of fine motor skills since childhood. The patient had inguinal hernia repair at around 12 months, bilateral hearing impairment with a left bone-anchored hearing aid, and spinal surgery. During spinal surveillance MPS VII was suspected by a spinal surgeon with interest in MPS, and the diagnosis confirmed with a deficiency in beta-glucuronidase in leucocytes and marginally elevated urinary GAGs. Next-generation sequencing identified two mutations in the *GUSB* gene (OMIM 611499), c.526C > T p.(Leu176Phe) and c.1820G > C p.(Gly607Ala). Although the patient exhibited features of the severe form of non-classical manifestations, his metabolic condition has remained reasonably stable, surviving into adulthood with only symptomatic treatment. We present the ever-expanding phenotypic spectrum of this ultra-rare disease.

## 1 Introduction

Mucopolysaccharidosis VII (Sly syndrome; MPS VII) (OMIM 253220) is an autosomal recessive disorder characterised by a deficiency in the enzyme Beta-glucuronidase (*GUSB;* Enzyme Commission (EC) number 3.2.1.31; MIM 611499). First described by Sly et al. in 1973 [1], MPS VII is linked to a mutation in the beta-glucuronidase gene located on chromosome 7q11.21-7q11.22. To date, >64 mutations have been identified [2].

The deficiency of GUSB enzymatic activity causes partial degradation of the following glucuronic acid-containing glycosaminoglycans (GAGs); chondroitin sulfate (CS), dermatan sulfate (DS) and heparan sulfate (HS), resulting in the accumulation of these fragments in the lysosomes of many tissues, eventually leading to multisystem damage.

The precise epidemiological data is scarce, and the estimated prevalence is 0.01 in 100,000 European live births [3]; and 0.027 in 100,000 in the United States [4]. Patients may not be diagnosed due to prenatal death [5]; but also misdiagnosed where hydrops fetalis is present and MPS VII is not suspected. The phenotype for MPS VII is variable, with some patients presenting with early, severe multisystem presentation (lethal non-immune hydrops fetalis) and others with late-onset manifestations surviving into the fifth decade of life [6].

In the overall presentation, MPS VII has many similar features to MPS I and MPS II. The most consistent features of MPS VII can include skeletal dysplasia, coarse facies with a short neck, pulmonary involvement, corneal clouding, cardiac valve disease, intellectual disability, hepatosplenomegaly; non-immune hydrops fetalis is commonly observed in severe cases [5,[7], [8], [9]]. It is also one of the few lysosomal disorders with a neonatal form with clinical manifestations in utero or at birth [[10], [11], [12], [13]].

Urinary GAGs measurement is the gold standard to screen for patients with MPS and a definitive diagnosis can be made by measuring GUSB enzyme activity. But as with the other MPS disorders, the help of an expert laboratory that uses both quantitative and qualitative methodology and age-corrected values is needed.

We report a case of a 31-year-old male patient diagnosed with MPS VII at the age of 28. Multiple specialists saw the patient over many years without suspecting the diagnosis due to unusual presentation.

## 2 Case presentation

The patient was the only child of healthy non-consanguineous parents with no history of disease on either side of the family. During the antenatal period, decreased fetal activity was noted, and the patient was born via caesarean section following a breech presentation. A neonatal exam showed congenital talipes on both feet and a single palmar crease across one hand. A brief period of strapping and physiotherapy rectified talipes. No history of hydrops fetalis was recorded.

Inguinal hernia repair was undertaken at around 12 months, and the family noticed a curvature of the spine around 18 months. An examination at the age of 2 indicated moderately severe pectus excavatum and marked scoliosis with prominence on the right side of the chest (both anteriorly and posteriorly). There was no indication of any gross neurological abnormality. The patient had a galaxy of dysmorphic features, a defect in the left ear lobe, and his fingers and thumbs were slightly unusual. At 9 years of age, complex thoracic deformity led to a rib strut grafting and an insertion of a third tubular plate, and significant scoliosis led to further spinal surgery.

There was a history of recurrent ear infections; and at the age of 13, the patient underwent myringoplasty surgery on the left side. At 17 years, a left single-stage bone-anchored hearing aid was implanted. At 20 years, the patient had a pinhole perforation of his right tympanic membrane, a left mastoid cavity, and was later diagnosed with chronic infection and cholesteatoma of the left ear.

The patient attended mainstream school however required a full-time support assistant. Unfortunately, formal cognitive assessments were not available from medical records. The patient completed skills for life at college but could not find any suitable job due to the combination of his physical disabilities and cognitive impairment.

Ophthalmologically, the patient was incorrectly diagnosed with corneal dystrophy as a teenager. The patient was also noted to be very photophobic. Ophthalmic surgeons suspected a metabolic condition and referred the patient to the metabolic clinic. During spinal surveillance, the spinal surgeon interested in MPS instigated an investigation at the age of 28 because of a bilateral corneal clouding diagnosis, slight coarse facial features, hearing loss, short stature, and significant scoliosis.

The patient was neurologically intact at presentation, had difficulty in the abduction of both shoulders, walking, and mobility had significantly deteriorated over time. The patient's muscle strength had reduced to around 3+/5 globally throughout the lower limbs. Since childhood, there has been a history of developmental delay, abnormal gait, and progressive deterioration of fine motor skills.

Laboratory analysis confirmed the MPS VII diagnosis with a deficiency in beta-glucuronidase in leucocytes (9 nmol/mg/h, reference range 100–800 nmol/mg/h) and marginally elevated urinary quantitative GAGs (8.2 mg/mmol (reference range 0–8 mg/mmol). Next-generation sequencing identified two mutations in the *GUSB* gene (OMIM 611499), c.526C > T p.(Leu176Phe) and c.1820G > C p.(Gly607Ala).

**Fig. 1:**
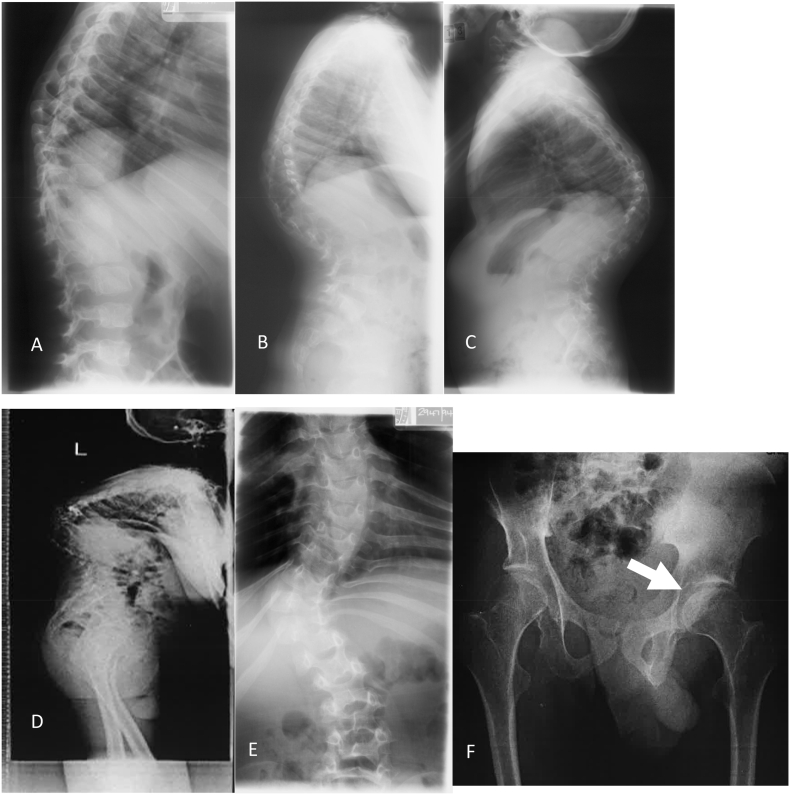
X-ray images of dysostosis multiplex showing progressive kyphosis at 4 years (A), 5 years (B), 7 years (C) and 28 years (D), significant scoliosis (E) and dysplastic hips (F), shallow hip sockets and femur head partly uncovered (arrow).

Further investigations including, abdominal ultrasound, showed no evidence of organomegaly. A computerised tomography (CT) scan of his neck showed spinal cord compression at his brain stem C1/C2 and slight instability. Neurological examination showed brisk reflexes in his upper limbs, including positive pectorals and Hoffman's. Respiratory function tests revealed reduced forced vital capacity, about 25% of normal and peak expiratory flow was also limited. X-ray imaging showed dysostosis multiplex (Fig. 1).

**Fig. 2:**
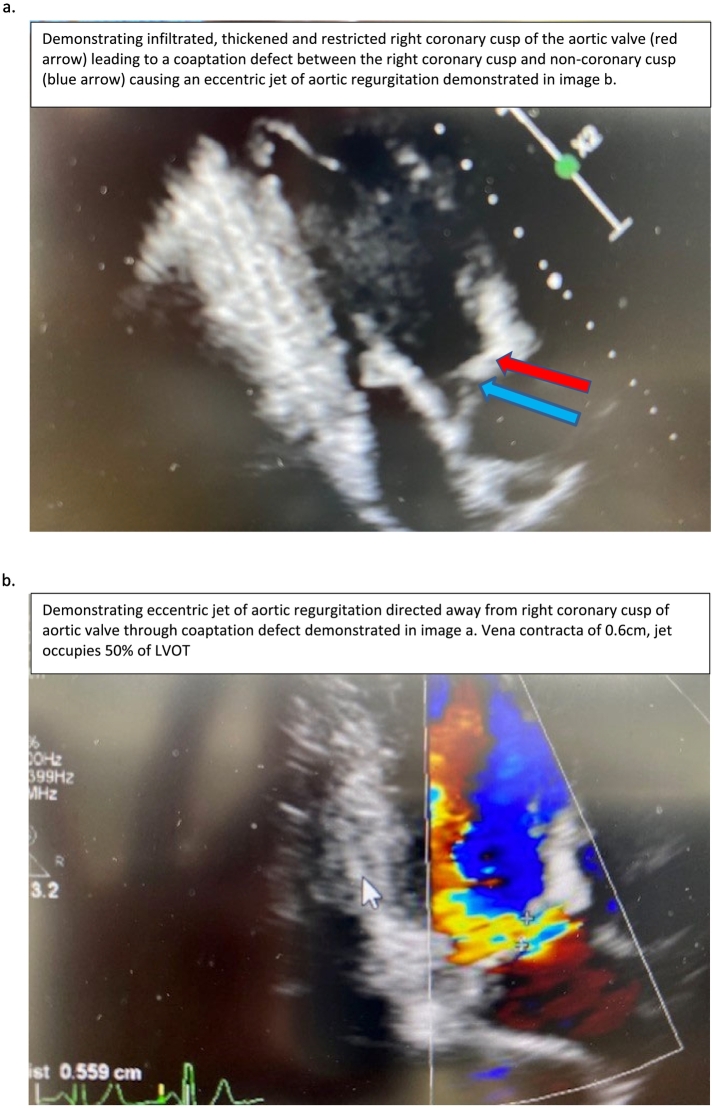

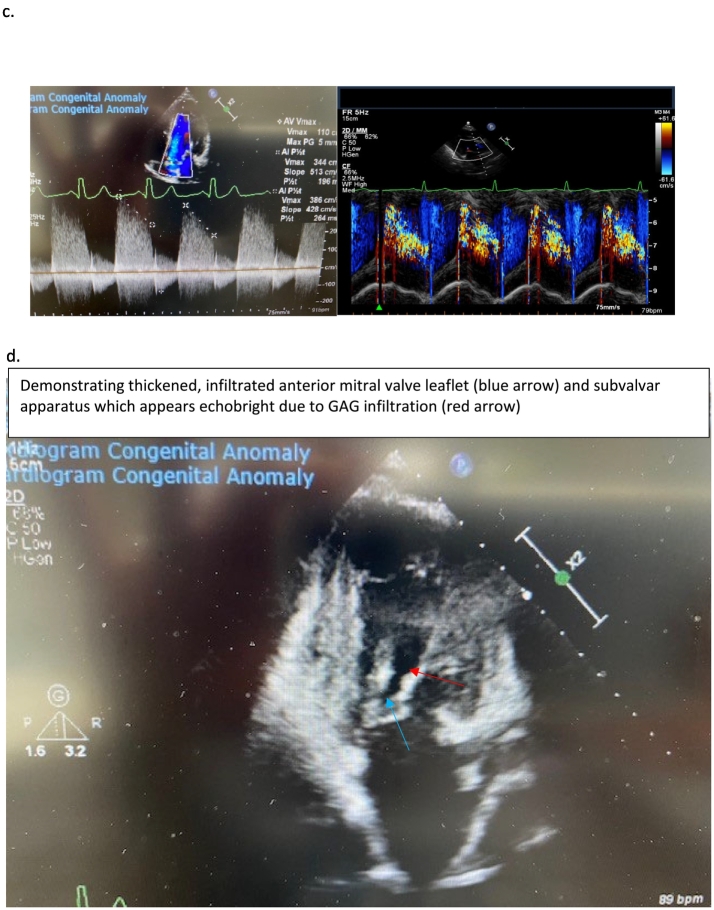
Echocardiogram features.

The echocardiogram (Fig. 2) demonstrated initially eccentric severe aortic regurgitation that improved to moderate with initiation and titration of angiotensin-converting enzyme inhibitors (ACEi). The right coronary cusp of the aortic valve was infiltrated with GAGs causing restriction of movement, a coaptation defect and therefore, eccentric jet of moderate aortic regurgitation. There was no aortopathy seen on the echocardiogram. The anterior mitral valve leaflet and subvalvular apparatus were infiltrated with GAGs. This led to restricted, tethered motion of the anterior mitral valve leaflet leading to significant mild mitral regurgitation.

A mildly dilated and impaired left ventricular (LV) systolic function improved to normal dimensions and function with ACEi. There were no regional wall motion abnormalities. It is unclear whether the initial LV dilation and impairment was secondary to the aortic regurgitation or represented underlying cardiomyopathy. Previous thoracic spinal surgery and the presence of the third tubular plate precluded the use of magnetic resonance imaging (MRI). The patient's right heart was unremarkable.

**Fig. 3:**
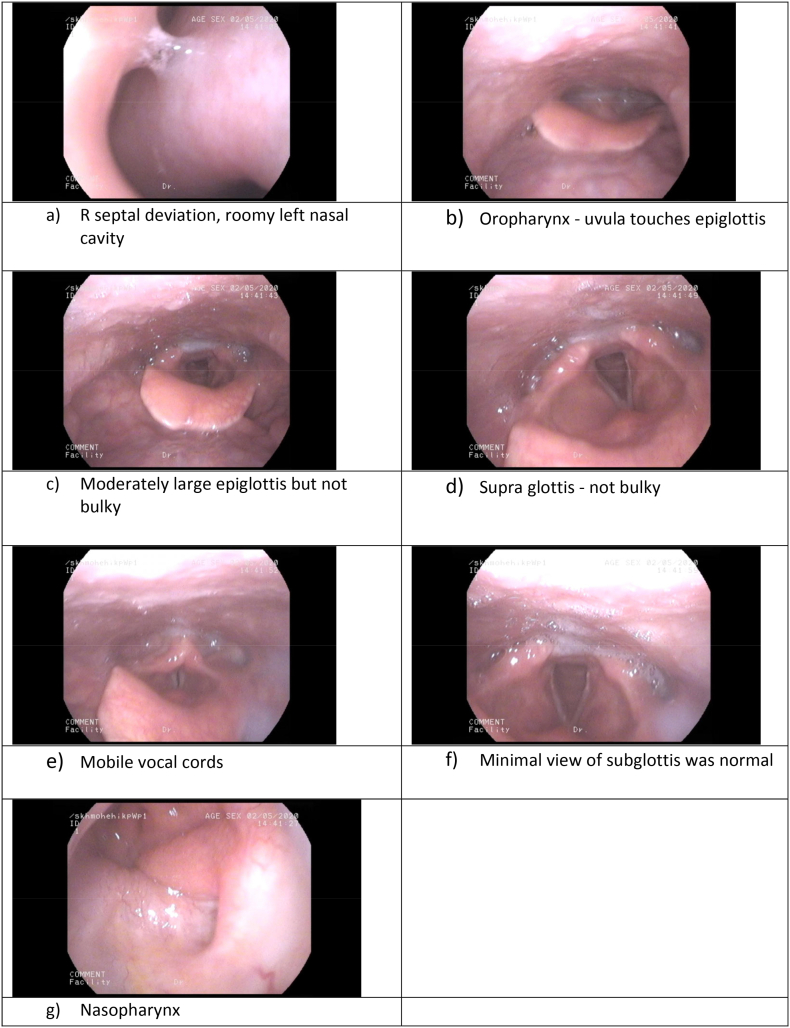
Flexible nasendoscopy revealed adequate nasopharynx, high larynx with hardly any distance between the epiglottis and tip of the soft palate. The supraglottis was not bulky, and both vocal cords were mobile. Minimal view of the subglottis appeared normal.

Following an airway assessment (Fig. 3), the patient reported no eating, drinking, or swallowing problems. There was no gastroesophageal reflux and no GAG deposits in the nose or nasopharynx. There are no records of difficulties around intubation with previous surgeries and the current airway assessment by clinical examination, endoscopy, cross-section imaging and 3-dimensional reconstruction support this. The patient's last anaesthetic was in 2011 for a minor ear nose and throat procedure; this was performed by laryngeal mask airway with pressure control ventilation. All the previous anaesthetic procedures including recovery were uneventful. Following assessment; bag-mask ventilation, laryngeal mask airway and intubation were deemed possible, however, front of neck access would be challenging due to short cervical trachea.

**Fig. 4:**
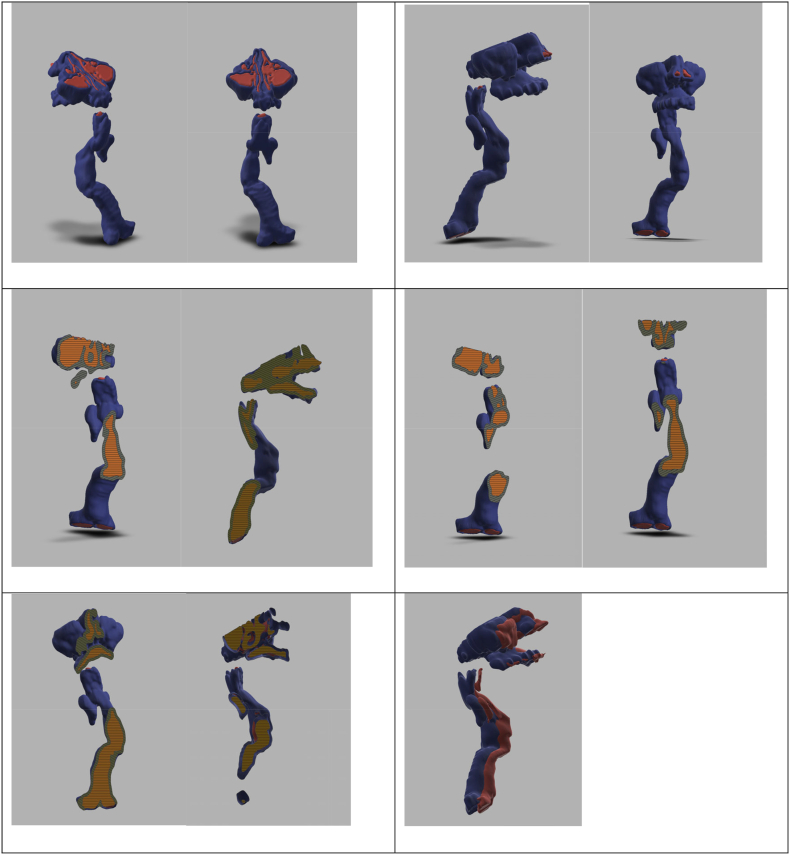
3-dimensional imaging of throat and trachea.

A computerised tomography scan revealed a slightly narrow subglottis of around 15 mm; the trachea became very tortuous, starting at the mid trachea and extending to the lower trachea; 3-dimensional reconstruction of the airways confirmed this (Fig. 4).

Although his metabolic condition has remained clinically stable over the last few years, there have been symptoms of decreased function in the upper and lower limb along with some uncontrolled movements. Weakening of the right leg has resulted in the patient requiring assistance to walk and use a wheelchair outdoors. He continues to attend regular physiotherapy sessions to prevent mobility deterioration. The patient's cardiac symptoms have remained stable, and there are no immediate plans for valve replacement.

The patient is currently not on enzyme replacement therapy. The disease-modifying treatment was explored; however, there was some reluctance from the family due to the patient's acceptable quality of life and relatively low burden of care on the family. The family's concerns around infusion-related reaction, time commitment, and travelling costs also contributed to their decision not to pursue enzyme replacement therapy.

## 3 Discussion and conclusion

Our patient presented with an intermediate or non-classical form of this ultra-rare lysosomal storage disorder, surviving into adulthood with only symptomatic treatment. The most frequent severe phenotype of MPS VII is non-immune hydrops fetalis, and other patients present with the non-classical form with late-onset manifestations. The patient did not have a history of hydrops fetalis, but a neonatal exam revealed congenital talipes of both feet and a single palmar crease across one hand. Montaño et al. found that approximately 35% of MPS VII patients presented with talipes equinovarus.

Multiple specialists saw the patient over many years without suspecting the diagnosis, highlighting how early diagnosis in some cases can be difficult on clinical grounds alone; due to how variable the clinical presentation and disease progression can be. Lack of awareness of ultra-rare diseases in mainline specialties contributed to the diagnostic odyssey which is frequently encountered by rare disease patients.

Literature on MPS VII reported cases have shown marked variation in phenotypic expression [5]. Patients who presented with mild/moderate manifestations had frequent respiratory infections, corneal clouding, coarse facial features with mild skeletal abnormalities. Those with the severe phenotype exhibited short stature, hepatosplenomegaly, recurrent ear infections, more significant skeletal abnormalities, hernias, macrocephaly and cognitive impairment [1,5,14,15]. The phenotypic heterogeneity of MPS disorders is a continuous spectrum, with some overlap often seen between subtypes as seen with this current patient. The non-classical form can present with mild symptoms in many organ systems or severe symptoms in one or two organ symptoms coupled with mild symptoms in other organ systems [12].

The patient exhibited the phenotypic features that indicated that he had the severe phenotypic presentation of MPS VII; however, the patient also demonstrated other features presented with mild/moderate manifestations suggesting a potential intermediate phenotype showing the wide heterogeneity of this disorder. At presentation, his abdominal ultrasound showed no evidence of organomegaly; however, his echocardiogram revealed cardiac involvement and severe aortic regurgitation. In the study by Montaño et al., 50% of MPS VII patients had cardiac valve disease and 37% presented with cardiomyopathies [5]. Additionally, 75% of patients had hepatomegaly/splenomegaly [5].

Ocular features can be an essential clue in diagnosing MPS VII [7,16]. The patient was noted to be photophobic and incorrectly diagnosed with corneal dystrophy at the age of 18. Corneal clouding was the predominant ocular feature in a study by Montaño et al. [5], observed in 63% of MPS VII patients. Although his corneal clouding has progressively worsened, he currently has a moderate reduction in vision (0.5 LogMar both) and requires glasses for hypermetropia. It is unclear whether the corneal clouding was present from a young age; however, the patient started wearing glasses at the age of 5. The patient has no symptoms of retinopathy.

The patient has remained neurologically stable for the last few years; however, he has shown some symptoms suggestive of myelopathy. CT imaging has confirmed the spinal cord compression at C1/C2. There is currently no adequate patient base to say whether the progression of kyphosis is associated with significant neurological deficits. Although kyphosis increases the incidence of neurological deficit in many MPS patients, the progressive deficit is rare. The patient had remarkably severe skeletal abnormalities from an early age that progressed with age (Fig. 1).

A cross-sectional analysis of 53 MPS VII patients found that residual GUS activity in fibroblasts >1.4% of normal controls had later onset of disease and lived longer than those with GUS enzymatic activity below this threshold [17]. They also found that urinary GAG excretion <602% of normal controls had longer survival than those with higher urinary excretion levels [17]. The patient's quantitative urinary GAGs were marginally elevated for age, with a small amount of dermatan sulphate present. At present, GUS activity in fibroblasts of the patient has not been investigated.

Determining severity using genotype-phenotype is limited due to the rarity of the disease; however, studies have shown a link between clinical severity and specific mutations [18]. Genetic analysis of the patient identified two heterozygous variants, c.526C > T p.(Leu176Phe) and c.1820G > C p.(Gly607Ala) missense mutations in the GUSB gene (OMIM 611499).

GUSB c.526C > T p.(Leu176Phe) is one of the most prevalent recurrent mutations for MPS VII and has been associated with the later-onset form of the disorder. GUSB c.1820G > C p.(Gly607Ala) has been reported in a patient with the severe form [18].

The rarity of MPS VII makes diagnostic suspicion difficult, as seen with this current patient; and in some cases, the precise diagnostic delay is unknown. The patient is one of the very few diagnosed with this ultra-rare disorder in the United Kingdom. Although he exhibited features of the severe form of non-classical manifestations, his metabolic condition has remained reasonably stable, surviving into adulthood with only symptomatic treatment.

Once diagnosed with MPS VII, the patient underwent a holistic airway and other systems assessment. This brought into light unreported airway abnormalities. Knowledge of these airway and cardiopulmonary abnormalities is vital as these patients may need airway intervention as a part of anaesthesia for any surgery for multisystem abnormalities. Our assessment pathway has helped us plan any intervention should the need arise [19].

The diagnostic odyssey for this patient is sadly not unique and awareness programs seemed to have made little difference. The lack of awareness of rare diseases, the overlap with more common disorders and the complexity of diagnostics cost, all contribute to these delays suggesting that potentially newborn screening and/or well-developed guidelines may be the best way forward. In this case, a skeletal panel may have been used in the disorder's early stages or the constellation of skeletal abnormalities, visual abnormalities and chronic otitis media could have been investigated with a urine GAG screen.

In conclusion, this case highlights the ever-expanding phenotypic spectrum of this ultra-rare disease. Index of suspicion and awareness amongst various health professionals regarding MPS is vital in early diagnosis, which may prevent irreversible deformities. A multidisciplinary approach is recommended involving multiple specialities and health professionals in this complex case led by an experienced metabolic medicine team.

## Details of funding

The work was supported by a medical grant from 10.13039/100013220Ultragenyx Pharmaceutical Inc. grant ID number HRG-OTH-941.

## Patient consent statement

The Father provided written consent on behalf of the patient for the publication of this case report.

## Conflict of interest

A.Oldham received speaker fees from FYMCA Medical Ltd. for a Pompe Masterclass. G.Tol received speaker fees/travel expenses from Biomarin Europe Ltd. J.Ashworth's institution received a grant from Biomarin. C.Hendriksz was employed by FYMCA Medical Ltd. until October 2020; currently, it is still family-owned; employed by Nestle Health Science since December 2020 but not related to this disease area. N.Oxborrow received speaker and consultation fees from Ethicon and Biomarin. P.Jenkins received travel expenses from Jansen. P.Woolfson received consultation fees for the MPS 1 advisory board in November 2020. A. Jovanovic declares no conflict of interests. M. Rothera declares no conflict of interests. C. Gadepalli received speaker fees/travel expenses from Biomarin Europe Ltd. A. Saxena declares no conflict of interests.

## Data Availability

No data was used for the research described in the article.
